# Mitochondrial Dysfunction in Renal Cell Carcinoma: A Comprehensive Review of Pathogenic Mechanisms and Emerging Therapeutic Opportunities

**DOI:** 10.32604/or.2026.082432

**Published:** 2026-08-13

**Authors:** Yanhong Wang, Junbo Liu, Qiaoping Xu, Zhao Ma

**Affiliations:** 1 Department of Pharmacy, the Second Affiliated Hospital, Zhejiang University School of Medicine, Hangzhou, China; 2 Department of Pharmacy, the Eighth Affiliated Hospital, Sun Yat-sen University, Shenzhen, China; 3 Department of Pharmacy, Affiliated Hangzhou First People’s Hospital, School of Medicine, Westlake University, Hangzhou, China; 4 Department of Pharmacy, Hangzhou Third People’s Hospital, Hangzhou Third Hospital Affiliated to Zhejiang Chinese Medical University, Hangzhou, China

**Keywords:** Kidney cancer, mitochondrial metabolism, oxidative phosphorylation, apoptosis evasion, mitochondrial quality control, targeted therapy, drug resistance

## Abstract

In renal cell carcinoma (RCC), alterations in cellular metabolism are a defining feature, among which impaired mitochondrial function stands out as a key factor influencing both tumor aggressiveness and patient responses to therapy. The aim of this review is to systematically synthesize current knowledge on the role of mitochondrial dysfunction in RCC pathogenesis and to explore emerging therapeutic strategies targeting mitochondrial vulnerabilities. This comprehensive analysis examines the integrated dysregulation of core mitochondrial processes—bioenergetic metabolism, organelle dynamics, programmed cell death pathways, redox homeostasis, and selective autophagy—in driving RCC pathogenesis. Our synthesis reveals how genetic drivers, molecular regulators, and microenvironmental cues converge to remodel mitochondrial function, creating both adaptive advantages and therapeutic vulnerabilities. A paradoxical duality emerges in mitochondrial biology: processes such as fission, mitophagy, and reactive oxygen species (ROS) generation can simultaneously support tumor adaptation while rendering cells susceptible to targeted interventions. We evaluate emerging therapeutic approaches directed at mitochondrial vulnerabilities, including metabolic inhibitors, nanoscale delivery systems, and phytochemical agents, while addressing current limitations in specificity and resistance mechanisms. Based on current preclinical evidence, this integrated perspective establishes mitochondrial dysfunction as a central determinant of RCC malignancy and suggests potential combinatorial strategies for precision oncology approaches that warrant further investigation.

## 1 Introduction

Renal Cell Carcinoma (RCC) persists as a major urological malignancy, representing a significant and often lethal threat to global health. It accounts for approximately 2–3% of all adult cancers, with its incidence notably rising over recent decades [1,2]. Epidemiological data from 2022 highlighted over 431,000 new RCC cases and nearly 180,000 deaths worldwide, underscoring its substantial disease burden [3]. A critical challenge in RCC management is the frequent absence of specific early-warning signs; a considerable proportion of patients are consequently diagnosed with advanced or metastatic disease, often discovered incidentally [4]. The ineffectiveness of conventional radiotherapy and chemotherapy against the most common histological subtype, clear cell renal cell carcinoma (ccRCC), complicates treatment, leaving surgical resection as the primary curative option for localized tumors [5]. Despite advancements in surgical techniques and the integration of targeted therapies (e.g., tyrosine kinase inhibitors) and immunotherapies (e.g., immune checkpoint inhibitors [ICIs]), outcomes for patients with advanced or metastatic RCC remain suboptimal, characterized by high risks of recurrence and the eventual development of therapy resistance [6]. The 5-year survival rate for patients with distant metastases remains disappointingly low, hovering below 15% [7]. This stark reality necessitates a deeper exploration of the molecular drivers of RCC pathogenesis to identify novel therapeutic vulnerabilities and improve clinical prognoses.

The development of RCC is influenced by a complex interplay of risk factors. Well-established determinants include smoking, obesity, hypertension, and certain hereditary genetic syndromes, such as von Hippel-Lindau (VHL) disease, which is intricately linked to the pathogenesis of sporadic ccRCC [7].

In recent years, mitochondria have emerged as important players in the pathogenesis and progression of RCC, extending far beyond their traditional role as cellular powerhouses. Mounting evidence underscores that mitochondrial dysfunction is a critical enabler of hallmark malignant behaviors in RCC, such as sustained proliferative signaling, invasive growth, metastatic dissemination, and resistance to conventional and targeted therapies [8,9].

However, an unresolved question persists in the field: under what conditions does mitochondrial dysfunction act as a driver versus a suppressor of RCC malignancy, and how do the five core mitochondrial processes—energy metabolism, dynamics, apoptosis, redox homeostasis, and mitophagy—differentially contribute to these opposing outcomes? From one vantage point, studies have illuminated how metabolic reprogramming and dynamic morphological shifts fuel the aggressive phenotype of RCC cells [10]. Conversely, other research highlights the potent tumor-suppressive capacity of mitochondria through apoptosis regulation. These ostensibly conflicting observations underscore the intricate and context-dependent nature of mitochondrial involvement in RCC.

To reconcile these controversies and provide a coherent conceptual framework, this article systematically synthesizes current literature on how five core mitochondrial processes—energy metabolism, dynamics, apoptosis, oxidative stress (OS), and mitophagy—are dysregulated in RCC. We further investigate how genetic alterations, key regulatory factors, and the tumor microenvironment (TME) converge to reshape mitochondrial function, and critically evaluate emerging therapeutic strategies targeting mitochondrial vulnerabilities, with the aim of providing insights for future clinical approaches for RCC management.

## 2 Overview of Mitochondria

Mitochondria are double-membraned organelles that carry their own genome (mitochondrial DNA, mtDNA) and function with a degree of independence from the nucleus. They house the oxidative phosphorylation (OXPHOS) machinery, whose core components—13 polypeptide subunits—are encoded by mtDNA [11]. Beyond their conventional role as adenosine triphosphate (ATP)—generating powerhouses, mitochondria in RCC have emerged as key signaling hubs that integrate metabolic inputs, trigger apoptosis, and sustain redox balance. Disruption of these functions is frequently observed during RCC progression [9,12,13].

**Figure 1:**
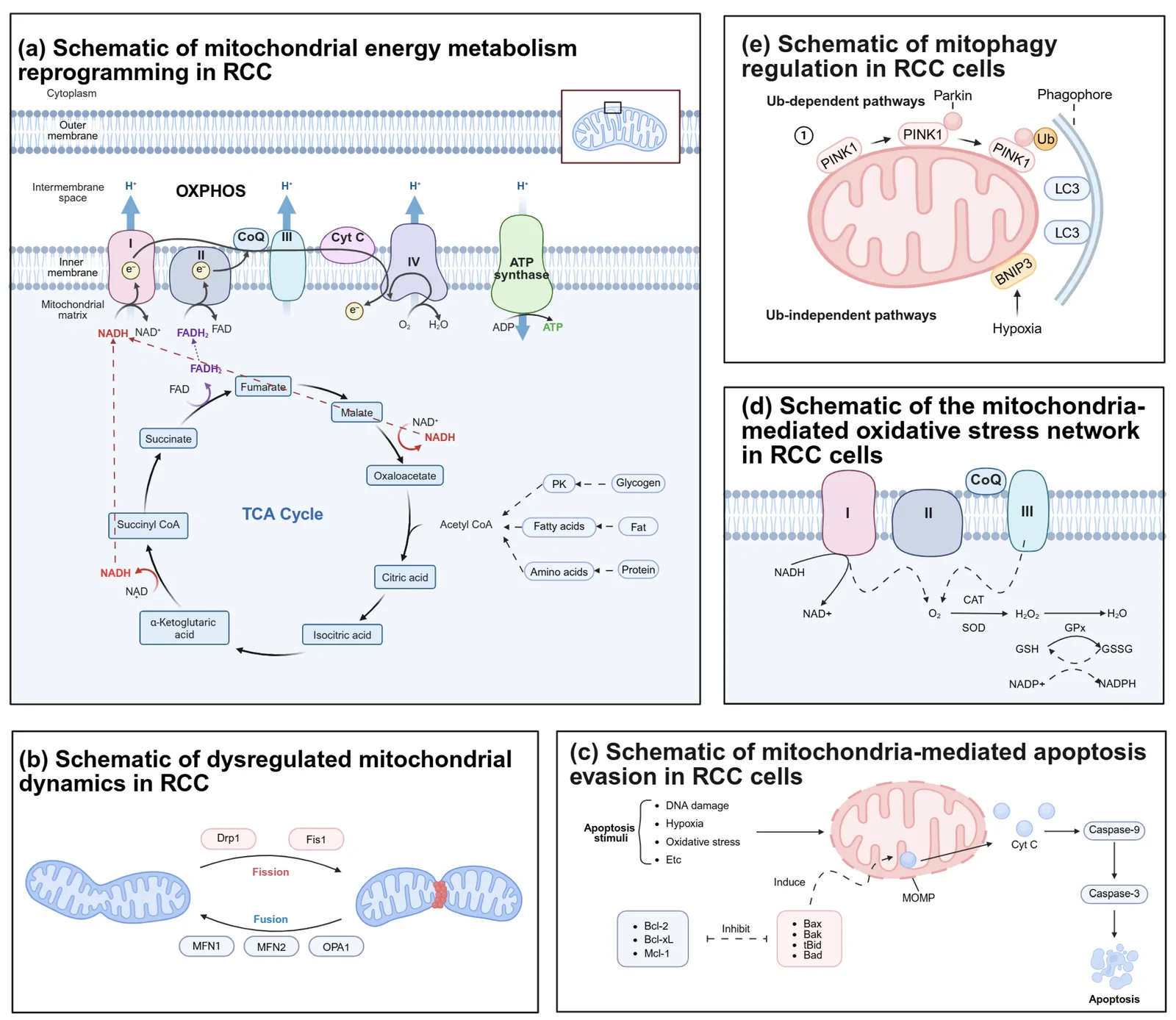
Schematic Illustrations of Five Key Mitochondrial Dysfunctions in Renal Cell Carcinoma (RCC). (**a**) Reprogramming of mitochondrial energy metabolism in RCC. (**b**) Dysregulated mitochondrial dynamics in RCC. (**c**) Evasion of mitochondria-mediated apoptosis in RCC cells. (**d**) Mitochondria-mediated oxidative stress (OS) network in RCC cells. (**e**) Regulation of mitophagy in RCC cells.

In RCC, mitochondrial bioenergetics is often rewired to fuel tumor growth, and mitochondrial morphology is highly adaptable, shaped by the continuous balance between fusion and fission [5,9,10]. These alterations span five interconnected domains: energy metabolism, dynamics, apoptosis, redox homeostasis, and mitophagy (summarized in Fig. 1).

Most current evidence on mitochondrial dysfunction in RCC comes from studies of ccRCC, the most prevalent subtype [5]. Limited data indicate that other subtypes, such as papillary RCC (pRCC) and chromophobe RCC (chRCC), may display distinct mitochondrial metabolic profiles. For example, pRCC has been linked to alterations in mtDNA and OXPHOS enzymes [14], while chRCC is characterized by abundant mitochondrial and ultrastructural abnormalities, including tubulovesicular cristae [15]. These subtype-specific differences remain poorly understood and warrant further investigation [16].

## 3 Mitochondrial Dysfunction: A Direct Driver of Malignant Progression in RCC

**Figure 2:**
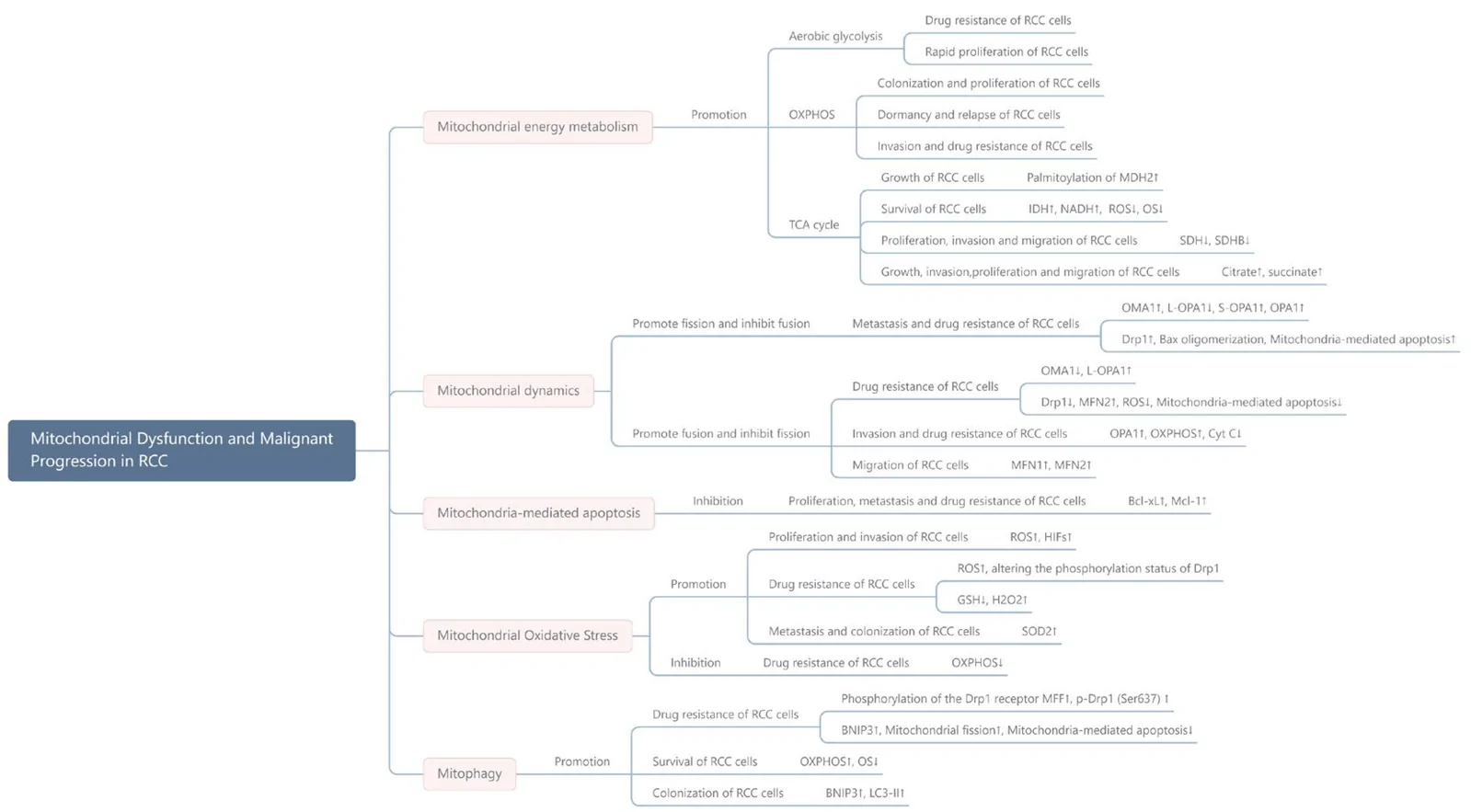
Mitochondrial dysfunction and malignant progression in RCC.

Mitochondria commonly exhibit profound functional impairments in RCC. In ccRCC, these defects are often initiated by VHL loss, which dysregulates hypoxic signaling and leads to metabolic reprogramming [17]. Beyond this canonical pathway, mitochondrial dysfunction in RCC can also stem from tricarboxylic acid (TCA) cycle enzyme deficiencies, electron transport chain (ETC) defects, persistent oxidative stress (OS), and mutations in both nuclear and mitochondrial DNA [18]. Collectively, these adaptive mechanisms fuel uncontrolled proliferation, metastatic dissemination, and therapy resistance (Fig. 2).

While the Warburg effect is a recognized feature, ccRCC maintains functional OXPHOS fueled by glutamine and fatty acids, particularly in advanced or treatment-resistant disease [18,19]. A shift toward glycolysis mitigates OS-induced damage, while resistance to Tyrosine Kinase Inhibitors (TKIs) is associated with enhanced OXPHOS dependency [20].

Metabolically distinct subpopulations—quiescent cells, stem cells, and invasive cells—frequently depend on OXPHOS fueled by fatty acid oxidation and glutamine metabolism [18,19]. During metastasis, transient metabolic shifts occur upon adhesion to secondary sites. Notably, studies using intraoperative 13C-labeled nutrient infusions in kidney cancer patients have demonstrated that ccRCC metastases unexpectedly exhibit enhanced TCA cycle labeling compared to primary tumors, indicating a divergent metabolic program during metastatic progression [21].

Dysregulation of TCA cycle enzymes is a hallmark of RCC. Enhanced palmitoylation of Malate Dehydrogenase 2 (MDH2) sustains mitochondrial respiration and promotes tumor growth [22]. Isocitrate Dehydrogenase (IDH) maintains TCA cycle flux; its inhibition depletes Reduced Nicotinamide Adenine Dinucleotide (NADH), increases ROS, and induces senescence [13]. Elevated IDH expression correlates with poor prognosis [23]. Succinate dehydrogenase (SDH) is frequently dysregulated; Succinate Dehydrogenase Subunit B (SDHB) deficiency leads to succinate accumulation, pseudohypoxia, and Epithelial-Mesenchymal Transition (EMT) [24]. IDH2 mutations have also been reported in aggressive RCC cases [25]. Furthermore, TCA intermediates can act as signaling molecules via retrograde signaling, propelling RCC development.

In summary, the available evidence indicates that metabolic reprogramming in RCC predominantly acts as a driver of malignancy by supporting tumor growth, metastasis, and therapy resistance.

## 4 Mitochondrial Dynamics

The equilibrium between mitochondrial fusion and fission is pathologically skewed in RCC, fostering adaptations that underpin tumor survival, metabolic plasticity, and disease progression [26,27].

A pro-fusion state, characterized by elongated mitochondria and altered cristae morphology, is associated with enhanced invasive potential and therapy resistance in RCC. Elevated Optic Atrophy 1 (OPA1) levels narrow cristae junctions, optimizing respiratory supercomplex assembly to boost OXPHOS efficiency while sequestering cytochrome c to raise the apoptotic threshold [28,29]. Overlapping Activity with m-AAA Protease 1 (OMA1) cleaves L-OPA1 to S-OPA1, inhibiting fusion and promoting fragmentation under stress. In RCC, the Stomatin-like Protein 2/Prohibitin 2 (STOML2/PHB2) complex suppresses OMA1 activity, preserving L-OPA1 and fostering a fused network that confers resistance to cell death [30,31]. Downregulation of Dynamin-related Protein 1 (Drp1) and upregulation of Mitofusin 2 (MFN2) have also been linked to therapy resistance, as this pro-fusion balance stabilizes mitochondrial membrane potential and attenuates ROS production, blunting apoptosis [32,33]. Nuclear receptors such as estrogen-related receptor α can induce MFN1/2 expression, facilitating EMT and metastasis [34].

Conversely, excessive fission is often a hallmark of apoptosis commitment, leading to network fragmentation, bioenergetic collapse, and release of apoptotic factors. In therapy-sensitive cells, genotoxic stress promotes p53 translocation to mitochondria, facilitating Drp1 activation and Bax oligomerization, culminating in fission and apoptosis [35]. In resistant cells, however, survival pathways such as PI3K-AKT inhibit Drp1, while OMA1-mediated OPA1 processing is suppressed, stabilizing the mitochondrial network and promoting survival [31,35].

Paradoxically, excessive fission also plays a pro-tumorigenic role in RCC. Drp1-driven fission supports cell survival and proliferation [36] and instigates a metabolic shift toward glycolysis [37]. This metabolic flexibility aids RCC cells in tolerating microenvironmental fluctuations [9,38]. Fission also facilitates invasion by accumulating fragmented mitochondria at the leading edge of migrating cells, localizing ATP production to fuel actin dynamics [39].

Mitochondrial fragmentation serves as an adaptive response to microenvironmental stress, functioning as a “metabolic circuit-breaker” that maintains viability under duress [26]. RCC cells can dynamically reverse this process at specific subcellular locations through integrin-mediated adhesion signaling, restoring elongated networks near focal adhesion sites to provide sustained energy for invasion [39].

Thus, mitochondrial dynamics in RCC exhibits a context-dependent duality: a pro-fusion state promotes survival and therapy resistance (driver), whereas excessive fragmentation can sensitize cells to apoptosis (potential suppressor under specific conditions) [33,36].

## 5 Mitochondria-Mediated Apoptosis in RCC

Evasion of mitochondria-mediated apoptosis constitutes a fundamental mechanism in the pathogenesis of RCC, enabling the survival and accumulation of genetically compromised cells. This apoptotic resistance primarily manifests through two interconnected mechanisms. First, a pronounced imbalance within the B-cell Lymphoma/leukemia-2 (Bcl-2) protein family is frequently observed in RCC, particularly the clear cell subtype. While Bcl-2 expression itself is variable, the consistent upregulation of potent anti-apoptotic members such as B-cell Lymphoma-extra Large (Bcl-xL) and myeloid cell leukemia-1 (Mcl-1) is considered a critical event that shifts the cellular equilibrium away from programmed cell death. This dysregulation may directly underpin the notorious resistance of RCC to conventional chemotherapy and targeted agents, with high levels of Mcl-1 and Bcl-xL being strongly correlated with advanced disease stage, metastatic progression, and inferior patient survival [40,41]. Second, the downstream apoptotic execution machinery is frequently impaired. Alterations in the expression and activity of executioner caspases disrupt the final stages of the cell death pathway, rendering RCC cells unresponsive to intrinsic pro-apoptotic signals [42]. Thus, it appears that the collective failure of the mitochondrial apoptotic pathway is intrinsically linked to the uncontrolled proliferation, metastatic dissemination, and profound treatment resistance that characterize advanced RCC [43,44].

Therefore, evasion of mitochondria-mediated apoptosis represents a well-established driver of RCC malignancy by enabling uncontrolled cell survival, proliferation, and resistance to therapy.

## 6 Mitochondrial OS in RCC

OS plays a context-dependent, dual role in the pathogenesis of RCC, critically influencing tumor behavior and therapeutic responses [38,45]. Mitochondria are the primary source of ROS in RCC cells, primarily through electron leakage from complexes I and III of the ETC [45,46]. The balance between ROS generation and elimination is frequently disrupted in RCC, contributing to malignant progression [47,48,49].

Building upon this network, OS exerts both pro-tumorigenic and therapy-modulating effects. OS may promote RCC proliferation, invasion, and therapy resistance through ROS-dependent cascades [50]. A key mechanism involves the hypoxic RCC microenvironment, where elevated ROS can activate Hypoxia-Inducible Factors (HIFs) even under normoxic conditions (pseudohypoxia), reinforcing oncogenic signaling [51]. Hypoxia-induced ROS also promotes adaptive changes such as Drp1 phosphorylation, inducing mitochondrial fragmentation associated with enhanced survival and TKI resistance [26]. 

The Glutathione (GSH) system plays a context-dependent dual role in RCC drug resistance. In the short term, elevated GSH levels detoxify chemotherapeutic agents and neutralize ROS, promoting cell survival and drug tolerance. Over prolonged exposure, sustained GSH consumption leads to depletion, impairing H_2_O_2_ scavenging and creating persistent oxidative stress that selects for resistant clones adapted to survive under high ROS conditions, ultimately exacerbating treatment failure [12].

Conversely, sustained ROS elevation can create therapeutic vulnerabilities. ROS may sensitize RCC cells by exacerbating DNA damage and disrupting mitochondrial membrane potential, lowering the death threshold for targeted therapies [50]. ROS is also a central executor of ferroptosis, an iron-dependent cell death distinct from apoptosis [52,53]. Ferroptosis is characterized by iron-dependent lipid peroxidation via the Fenton reaction, executed through Glutathione Peroxidase 4 (GPX4) inactivation or system Xc-inhibition [53,54]. Cancer cells with high iron metabolism, including ccRCC, exhibit increased ferroptosis sensitivity [55]. Research in RCC indicates that hydroxyl radicals generated via the Fenton reaction induce lethal lipid peroxidation, suppressing tumor proliferation and invasion. Importantly, targeting ferroptosis may overcome therapy resistance, as mesenchymal therapy-resistant cells often display enhanced ferroptosis susceptibility due to dysregulated iron homeostasis [56]. This highlights ferroptosis induction as a potential RCC therapeutic strategy. Notably, RCC tumors with high OXPHOS activity may develop chronic low-level OS, rendering them more susceptible to agents that further disrupt metabolic homeostasis [9].

During metastasis, RCC cells exhibit precise ROS regulation. Upon encountering microenvironmental stress, upregulation of Superoxide Dismutase 2 (SOD2) represents a critical early event for metastatic success. Located within mitochondria, SOD2 converts superoxide to H_2_O_2_, alleviating oxidative burden and facilitating survival and colonization of distant organs [57,58]. Successful metastatic RCC cells must maintain ROS within a narrow optimal window—leveraging ROS for pro-proliferative signaling while avoiding thresholds that induce senescence, apoptosis, or ferroptosis [53,59].

In summary, ROS functions as a double-edged sword in RCC: low-to-moderate ROS levels activate adaptive survival pathways and drive tumor progression (driver), whereas excessive ROS overwhelms antioxidant defenses, inducing irreversible damage and cell death (suppressor).

## 7 Mitophagy

In RCC, mitophagy can function as a tumor-suppressive mechanism by eliminating dysfunctional mitochondria. This quality control process is tightly coupled to mitochondrial dynamics, as fission-mediated fragmentation is a prerequisite for efficient autophagic recognition and clearance [1]. The PINK1-Parkin axis and Drp1 centrally regulate this pathway, exerting significant influence over RCC progression and therapeutic responses [60,61].

For instance, targeting the CRL4^CUL4A/DDB1^ E3 ubiquitin ligase complex activates the PINK1-Parkin pathway in RCC models, inducing Mitochondrial Fission Factor (MFF) phosphorylation and Drp1 dephosphorylation at Ser637, enhancing fission activity. Activated Drp1 interacts with Voltage-Dependent Anion Channel 1, triggering extensive fission and augmenting mitophagic clearance, ultimately suppressing RCC proliferation and restoring chemosensitivity [62].

Mitophagy demonstrates a context-dependent dual role in RCC, serving as a critical survival mechanism under stressful conditions. Under hypoxic and nutrient-deprived conditions characteristic of RCC, mitophagy is upregulated, promoting tumor cell survival by eliminating damaged organelles and maintaining a healthy mitochondrial pool [63,64]. This process is closely linked to therapy resistance: hypoxia-induced Hypoxia-Inducible Factor 1-alpha (HIF-1α) stabilization in resistant cells upregulates BNIP3, driving excessive fission and clearance, reducing cytochrome c release, and blocking apoptosis [56]. Consequently, inhibiting mitophagy presents a promising strategy for treatment-resistant RCC cells. Resistant RCC cells typically exhibit a more pronounced oxidative metabolic profile, relying on efficient mitophagic turnover to sustain functional mitochondrial populations [9]. During metabolic transitions such as reoxygenation or Extracellular Matrix adhesion, Bcl-2/adenovirus E1B 19-kDa Interacting Protein 3 (BNIP3) and Microtubule-associated Protein 1 Light Chain 3-II (LC3-II) expression is upregulated, supporting RCC cell survival during colonization via synergistic interactions with Peroxisome Proliferator-activated Receptor Gamma Coactivator 1-alpha (PGC-1α) [65]. Pro-tumorigenic macrophages in the TME also utilize mitophagy to alleviate their own oxidative stress, maintaining high OXPHOS activity and creating a supportive niche that enhances cancer cell survival under cytotoxic stress [66].

Similar to other malignancies, the Parkin/PINK1-dependent mitophagy pathway demonstrates tumor-suppressive potential in RCC by eliminating dysfunctional mitochondria and modulating HIF-1α/ROS signaling [67]. BNIP3 displays a stage-dependent expression pattern, typically upregulated in early disease but downregulated in advanced RCC [68]. Distinctive features of mitophagy regulation in RCC include: (1) synergistic interaction between constitutive HIF-1α stabilization (from VHL loss) and BNIP3-mediated mitophagy promoting survival under therapeutic stress, contributing to TKI resistance [69]; (2) unique cellular crosstalk within the RCC TME, particularly with lipid-laden macrophages and Cancer-Associated Fibroblasts, creating specialized niches that modulate mitophagic activity [70,71]; (3) dynamic regulation during metabolic transition phases supporting colonization competence [68]. These RCC-specific characteristics position mitophagy as a promising therapeutic target for overcoming treatment resistance and suppressing metastasis, though further *in vivo* and clinical studies are required [67].

Thus, mitophagy displays a dual role in RCC: transient, low-level mitophagy serves a homeostatic function and supports cell survival under stress (driver), whereas persistent or excessive mitophagy may deplete the mitochondrial pool beyond a viable threshold, leading to cell death (potential suppressor).

## 8 Upstream Regulatory Mechanisms Contributing to Mitochondrial Dysfunction in RCC

**Figure 3:**
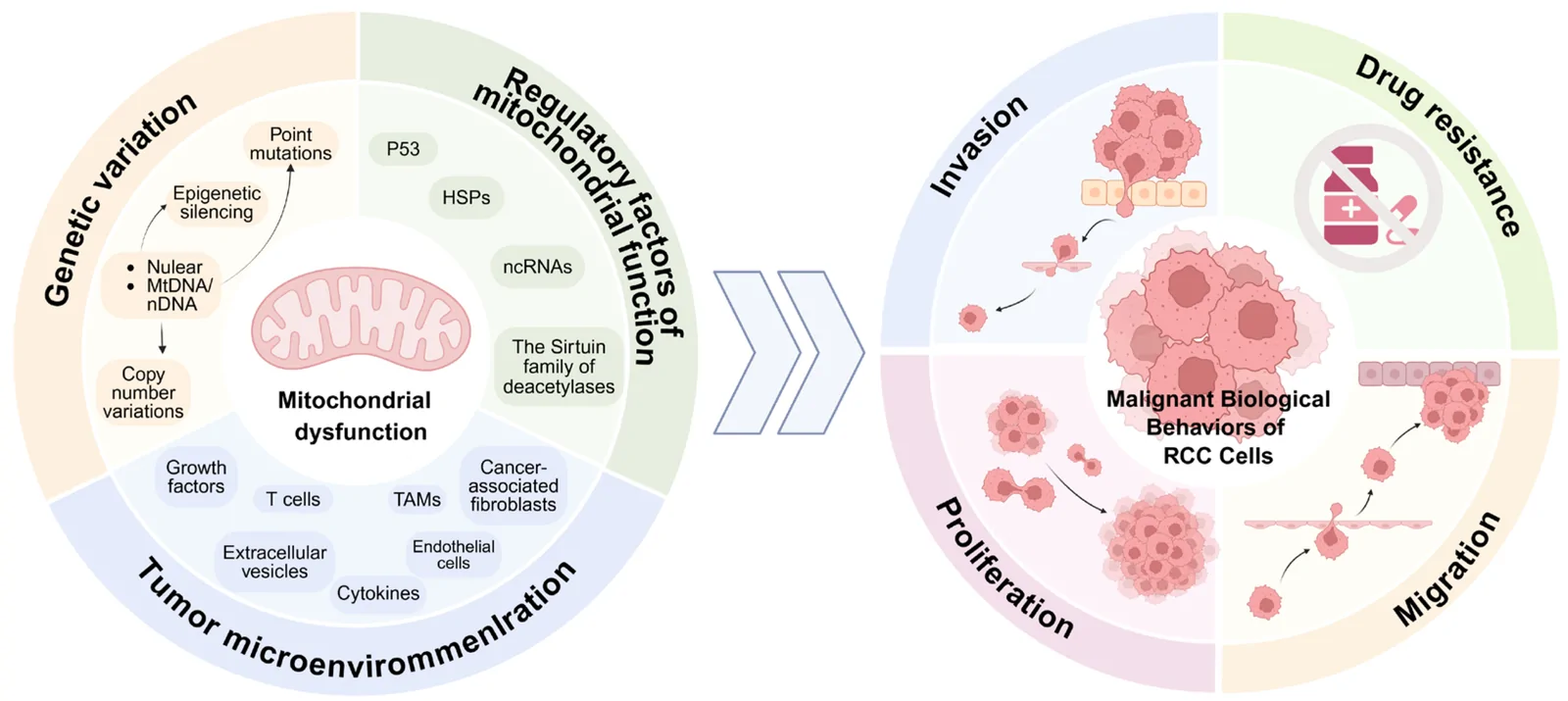
The Major Mechanisms of Mitochondrial Dysregulation in RCC Progression. This figure summarizes three upstream mechanisms driving mitochondrial dysfunction in RCC: (1) genetic/epigenetic alterations (nuclear DNA (nDNA) mutations, copy number variations, epigenetic silencing, and non-coding RNAs (ncRNAs)); (2) regulatory factors (p53, heat shock proteins (HSPs), and sirtuins); and (3) the tumor microenvironment (T cells, tumor-associated macrophages (TAMs), cancer-associated fibroblasts, endothelial cells, growth factors, cytokines, and extracellular vesicles). All three converge on mitochondrial dysfunction, promoting malignant behaviors such as proliferation, metastasis, and therapy resistance.

This section explores how aberrant mitochondrial activity in RCC drives tumor malignancy through three principal pathways: genetic alterations, regulatory factors, and the TME (Fig. 3).

### 8.1 Genomic Contributions to Mitochondrial Dysfunction

The genetic landscape of RCC is characterized by a complex constellation of alterations, including point mutations in both nuclear and mtDNA (nDNA and mtDNA), copy number variations, and epigenetic silencing. These modifications induce significant structural and functional remodeling of mitochondria, ultimately driving RCC tumorigenesis and progression by influencing cancer cell metabolism, proliferation, invasiveness, and therapy responses. 

#### 8.1.1 mtDNA Copy Number Variations

Alterations in mtDNA copy number represent a key genetic event in the pathogenesis of RCC [72,73]. Circulating cell-free mtDNA levels in RCC patients’ plasma change significantly following nephrectomy or targeted therapy, underscoring the dynamic role of mtDNA as a potential biomarker [74,75]. Mechanistically, enhanced mitochondrial biogenesis—reflected by increased ETC activity and citrate synthase levels—correlates with elevated mtDNA content in RCC tissues [51]. Genomic analyses further reveal specific alterations in SDH subunits in certain RCC subtypes, with SDHB mutations or loss of expression being a hallmark of hereditary RCC [16].

MtDNA content demonstrates considerable intratumoral heterogeneity in RCC, mirroring the metabolic reprogramming and variable proliferative rates across different tumor regions [76,77]. Paradoxically, some aggressive RCC variants exhibit lower average mtDNA copy numbers than less aggressive ones [72]. This may reflect that low-grade tumors engage compensatory mtDNA biogenesis to maintain energy production, while high-grade, rapidly proliferating tumors accumulate mitochondrial OS, leading to mtDNA damage and eventual depletion [72,78,79].

#### 8.1.2 mtDNA Mutations

Beyond alterations in copy number, mutations and genomic instability of mtDNA are increasingly recognized as critical factors in the pathogenesis and progression of RCC. Comprehensive sequencing analyses indicate a high mutational burden in the mitochondrial genome of RCC tissues compared to adjacent normal kidney tissue, with frequent somatic mutations identified in key regions including the D-loop regulatory region, rRNA genes (12S and 16S), and protein-coding genes such as Cytochrome b [80]. These mutations, often characterized by specific transition patterns, may contribute to the acquisition of aggressive traits, including enhanced stemness and metastatic potential in RCC cells [81]. The role of mtDNA mutations in RCC metastasis is supported by studies analyzing different biological samples. For instance, the detection landscape of specific mtDNA mutations can differ between primary tumor tissue, peripheral blood, and urine sediments in RCC patients, reflecting distinct routes of disease dissemination and offering non-invasive diagnostic potential [82]. Notably, certain mutation signatures, such as an increased ratio of T > C transitions, have been associated with metastatic disease, suggesting their utility as molecular markers of tumor progression [82]. Evolutionary analysis of mtDNA in RCC reveals a complex, adaptive pattern. To preserve essential respiratory function, protein-coding genes critical for OXPHOS, particularly those in Complex V (ATP6/ATP8), often undergo negative (purifying) selection, minimizing deleterious mutations. Conversely, mutations in genes encoding for Complex III subunits may be under positive selection, potentially conferring a metabolic advantage that supports tumor survival under stress conditions, such as hypoxia [83,84]. A broader profiling study in ccRCC identified numerous mtDNA variants affecting tRNA genes and OXPHOS components (e.g., COI, COIII, ATP6), which are postulated to alter energy metabolism and fuel malignant behavior, correlating with adverse patient outcomes [80]. Furthermore, mtDNA mutations are implicated in treatment resistance. In metastatic RCC patients receiving TKI therapy, the pattern of mtDNA mutations may differ between responding and non-responding cases. The presence of non-synonymous (missense) mutations, which can impair respiratory chain efficiency and promote a glycolytic shift, has been linked to a higher likelihood of therapeutic resistance and disease recurrence, highlighting their role in driving adaptive metabolic remodeling [44,85].

#### 8.1.3 nDNA Genetic Alterations

The phenotypic aggressiveness of RCC is fundamentally shaped by nuclear genetic alterations that control mitochondrial activity. nDNA-encoded proteins dictate the metabolic and signaling functions of mitochondria, thereby determining key pathological features of RCC, including its characteristic metabolic reprogramming and propensity for metastasis [86].

In ccRCC, the metabolic landscape is profoundly shaped by alterations in nuclear DNA-encoded mitochondrial regulators. For instance, the Mitochondrial Pyruvate Carrier responsible for pyruvate import, is frequently downregulated due to VHL loss and HIF activation [85]. This loss impedes the flux of pyruvate into oxidative phosphorylation, disrupting normal metabolic cycles and promoting a glycolytic phenotype that supports tumor growth and invasion [83]. Concurrently, inactivating mutations in genes such as ARID1A and PBRM1, key subunits of the SWI/SNF chromatin-remodeling complex found in a significant subset of RCCs, lead to significant mitochondrial rewiring [9,79]. These genetic alterations are associated with changes in mitochondrial morphology and function, often coupled with stabilization of the oncoprotein cellular myelocytomatosis oncogene, which further drives proliferative metabolism [9,83]. The dependency of such tumors on specific metabolic pathways creates vulnerabilities; for example, tumors with distinct metabolic signatures may show heightened sensitivity to inhibitors of mitochondrial complex I [83]. Beyond central carbon metabolism, variations in genes governing mitochondrial dynamics and OS response are also implicated in influencing RCC pathogenesis, prognosis, and response to therapeutic agents [9,83].

#### 8.1.4 Epigenetic Regulation

Epigenetic modifications—including DNA methylation, histone post-translational modifications, and non-coding RNA networks—are pivotal regulators of tumor cell behavior, often exerting their effects through mitochondrial reprogramming [87]. The ten-eleven translocation family of methylcytosine dioxygenases, which are frequently dysregulated in RCC, can enhance mitochondrial biogenesis and OXPHOS capacity by mediating demethylation of nuclear genes encoding mitochondrial proteins [88]. This process, potentially involving alterations in metabolites like α-ketoglutarate (α-KG), may modulate mitochondrial complex activity and energy production, influencing tumor cell fitness under specific microenvironmental conditions. Conversely, promoter hypermethylation is a common mechanism for silencing critical tumor suppressors in RCC. The loss of genes such as VHL (a hallmark of ccRCC) or other factors disrupts mitochondrial metabolism, stabilizing HIF-α subunits and driving a potent glycolytic shift that promotes progression and therapeutic resistance [89]. The histone methyltransferase EZH2, which catalyzes the repressive H3K27me3 mark, is overexpressed in aggressive RCC, silencing differentiation and tumor suppression genes [90]. Additionally, long non-coding RNAs (lncRNAs) contribute to RCC pathogenesis. Some lncRNAs can form regulatory axes that suppress mitochondrial apoptosis pathways (e.g., by influencing Bcl-2/Bax balance), thereby fostering cell survival and conferring resistance to systemic therapies [91,92]. 

### 8.2 Regulatory Factors Shaping Mitochondrial Activity

Mitochondrial function is orchestrated by a complex interplay of regulatory factors beyond genetic alterations. These multi-layered mechanisms—encompassing epigenetic, transcriptional, and metabolic adaptations—ultimately converge to dictate RCC cell fate [83,93]. Critical modulators include non-coding RNAs (ncRNAs), p53, heat shock proteins (HSPs), and the Sirtuin family, which often interact with canonical pathways like the VHL-HIF axis.

#### 8.2.1 ncRNAs in Mitochondrial Regulation

ncRNAs constitute the vast majority of the human transcriptome and are key players in RCC pathogenesis. They are broadly classified into miRNAs, lncRNAs, and circRNAs [94]. miRNAs post-transcriptionally regulate gene expression, targeting a large proportion of protein-coding genes involved in metabolism and apoptosis [95,96]. lncRNAs participate in transcriptional regulation and chromatin remodeling. circRNAs, with stable closed-loop structures, function as post-transcriptional regulators [97]. Many lncRNAs and circRNAs act as ceRNAs or “sponges,” sequestering miRNAs and attenuating their repression of target genes [98].

In RCC, a network of ncRNAs exerts precise control over mitochondrial function to influence tumor behavior. For instance, the tumor suppressor miR-145 is frequently downregulated in RCC. Its restoration has been shown to inhibit cancer cell proliferation by targeting key mitochondrial proteins, potentially reducing mitochondrial membrane potential (MMP) and promoting Cytochrome c release, thereby impairing energy metabolism and inducing apoptosis [99]. Conversely, oncomiRs such as the miR-221/222 cluster are upregulated and promote RCC progression by enhancing OXPHOS and suppressing apoptotic pathways, thereby boosting tumor cell survival and invasion [100]. Similarly, the downregulation of miR-340 in RCC contributes to metabolic reprogramming by failing to restrain the expression of mitochondrial metabolic enzymes, facilitating a pro-tumorigenic shift in energy production [101]. Among long non-coding RNAs, LINC00460 is highly expressed in ccRCC and acts as a molecular sponge for miR-149-5p, leading to increased expression of its target gene MYB Proto-oncogene, transcription factor (MYB). This axis promotes mitochondrial biogenesis and OXPHOS, fueling RCC growth [102]. In contrast, the lncRNA MEG3 functions as a tumor suppressor. Its expression induces mitochondrial apoptosis by modulating the Bax/Bcl-2 ratio and caspase activity, thereby inhibiting RCC proliferation and migration [103]. Furthermore, the lncRNA SNHG12 drives Warburg effect by disrupting normal mitochondrial metabolism, highlighting how ncRNAs can rewire central carbon metabolism in RCC [104]. Circular RNAs also play a role; for example, circ-AKT3 has been reported to inhibit glycolysis and promote mitochondrial respiration in RCC, influencing the metabolic phenotype of the tumor [105]. These findings collectively underscore the pivotal role of ncRNAs in regulating mitochondrial dynamics and metabolism in RCC (Table 1).

**Table 1:** Non-coding RNAs involved in mitochondrial regulation of renal cell carcinoma.

ncRNA	Targets/Interacting Molecules	Affected Mitochondrial Function	Impact on RCC Malignant Behavior	References
miR-145	ABHD4, HEXIM1	Energy Metabolism ↓, Apoptosis ↑	Inhibits	[106]
miR-221/222	PTEN, p27	Energy Metabolism ↑, Apoptosis Resistance ↑	Promotes	[100]
miR-340	PKM2	Energy Metabolism ↑ (Warburg Effect)	Promotes (when downregulated)	[101]
LINC00460	miR-149-5p/MYB	Energy Metabolism ↑ (OXPHOS)	Promotes	[102]
MEG3	miR-21/PTEN, Bax/Bcl-2 Ratio	Apoptosis ↑	Inhibits	[103]
SNHG12	miR-129-5p/PKM2	Energy Metabolism ↑ (Warburg Effect)	Promotes	[104]
circ-AKT3	miR-516b-5p/PDK1	Energy Metabolism ↓ (Glycolysis ↓, OXPHOS ↑)	Inhibits	[105]

Abb: ncRNA, non-coding RNA; miR, microRNA; ABHD4, abhydrolase domain containing 4; HEXIM1, hexamethylene bis-acetamide inducible 1; PTEN, phosphatase and tensin homolog; p27, cyclin-dependent kinase inhibitor 1B; PKM2, pyruvate kinase M2; MYB, MYB proto-oncogene, transcription factor; PDK1, pyruvate dehydrogenase kinase 1; Bax, Bcl-2-associated X protein; Bcl-2, B-cell lymphoma/leukemia-2; LINC00460, long intergenic non-protein coding RNA 460; MEG3, maternally expressed gene 3; SNHG12, small nucleolar RNA host gene 12; circ-AKT3, circular RNA AKT3; OXPHOS, oxidative phosphorylation.

#### 8.2.2 Additional Regulatory Factors (PGC-1α, HIF-1α, PPARα, CPT1A)

Metabolic reprogramming is a hallmark of RCC, driven significantly by alterations in mitochondrial function and substrate utilization. As a master regulator of mitochondrial biogenesis, PGC-1α is implicated in enhancing OXPHOS in certain RCC subtypes. This metabolic shift increases the reliance on mitochondrial energy production, potentially facilitating evasion of cell death and supporting tumor survival. The canonical metabolic profile of ccRCC is orchestrated by the HIF-1α pathway. Stabilization of HIF-1α promotes glycolytic metabolism and suppresses OXPHOS, a key mechanism driving RCC progression and resistance to therapeutic agents [18,107]. Conversely, strategies to knock down or degrade HIF-1α can redirect metabolic flux back towards mitochondrial OXPHOS, which has been shown to re-sensitize cancer cells to apoptosis and cytotoxic drugs [108]. The Peroxisome Proliferator-Activated Receptor Alpha (PPARα) pathway also contributes to the metabolic plasticity of RCC. The PPARα inhibitor GW6471 was shown to significantly suppress oxygen consumption in renal cancer models. This inhibition reduces the cellular dependence on fatty acids, glucose, and glutamine for energy, leading to suppressed tumor growth *in vivo*, underscoring PPARα‘s role in regulating RCC metabolism via mitochondrial activity [109]. Furthermore, fatty acid metabolism is critically involved. Carnitine Palmitoyltransferase 1A (CPT1A), the rate-limiting enzyme for mitochondrial fatty acid β-oxidation, promotes the succinylation of MFF at a specific lysine residue. This post-translational modification shields MFF from Parkin-mediated ubiquitination and proteasomal degradation. The subsequent stabilization of MFF protein levels enhances mitochondrial fission, a process that accelerates tumor proliferation and progression in RCC [9,110].

#### 8.2.3 Interplay among p53, HSPs, and Sirtuins

p53 promotes mitochondrial dysfunction; its activation has been linked to the suppression of key mitochondrial chaperones like HSPA9, while also inducing pro-apoptotic mitochondrial peptides. Conversely, HSP70 preserves mitochondrial integrity and counteracts p53-mediated cell death by inhibiting Bax activation [111]. The metabolic landscape of RCC is further shaped by a delicate balance between sirtuins and molecular chaperones. SIRT3, a major mitochondrial deacetylase, acts as a metabolic checkpoint by promoting OXPHOS and sensitizing cells to apoptosis [112]. In opposition, the mitochondrial chaperone TRAP1 fosters a glycolytic state, even under nutrient stress, by repressing OXPHOS and suppressing mitochondrial respiration, thereby enhancing tumor cell survival [113]. Adding another layer of regulation, non-coding RNAs directly target mitochondrial components. miR-145, a frequently downregulated tumor suppressor in RCC, has been shown to impair mitochondrial function and suppress cell proliferation by directly targeting and downregulating the expression of mitochondrial proteins such as HSP60, leading to disrupted OXPHOS [99]. These intricate interactions suggest that simultaneously targeting multiple nodes (e.g., combining a TRAP1 inhibitor with a SIRT3 activator) might be more effective than single-target interventions.

### 8.3 The TME as a Modulator of Mitochondrial Function

The RCC TME possesses distinctive characteristics that play an essential role in disease development and progression [114]. This ecosystem comprises not only cancer cells but also a heterogeneous population of stromal and immune elements, including tumor-associated macrophages (TAMs), T lymphocytes, cancer-associated fibroblasts, and endothelial cells [18,115]. In addition to these cellular components, the acellular fraction contains abundant cytokines, growth factors, and extracellular vesicles that enable intercellular signaling. A primary mechanism through which the TME drives RCC malignancy involves the induction of mitochondrial and metabolic adaptation, thereby promoting cancer cell survival, proliferation, and invasive capacity [114,115].

RCC, particularly ccRCC, is characterized by a high propensity for metastatic spread to distant organs such as lung, bone, and liver. Emerging evidence indicates that stromal cells within the tumor niche, including cancer-associated fibroblasts, can enhance tumor heterogeneity and metastatic capacity through the intercellular transfer of mitochondria to RCC cells exhibiting mitochondrial dysfunction. This mitochondrial donation phenotypically rescues the recipient cancer cells, reinstating their proliferative capacity, chemoresistance, and oxidative phosphorylation [116]. The RCC TME, rich in lipids and cytokines, inflicts oxidative damage upon surrounding endothelial and normal renal tubular cells, triggering mitochondrial superoxide overproduction, reducing mitochondrial membrane potential, and upregulating PGC-1α activity. These alterations collectively stimulate cancer cell proliferation and invasive behavior [117]. TAMs in RCC display remarkable metabolic plasticity in response to local cues, shifting from a glycolytic, pro-inflammatory (M1-like) phenotype to an OXPHOS-dependent, immunoregulatory (M2-like) state that supports tumor growth. M2-polarized TAMs often demonstrate increased mitochondrial mass and elevated oxygen consumption rates, which are instrumental in their tumor-promoting functions [118,119]. MtDNA released into the circulation or tumor interstitial fluid serves as a potential biomarker and active player in the RCC TME. It acts as a damage-associated molecular pattern that can trigger pro-inflammatory responses in immune cells like neutrophils. Elevated levels of circulating mtDNA are associated with advanced disease stage and poorer survival outcomes in RCC patients, potentially by fostering a pro-metastatic environment and compromising anti-tumor immunity [120].

Extracellular vesicles (EVs) have been reported to play a pivotal role in the progression of RCC by mediating intercellular communication through the transfer of mitochondrial constituents, nucleic acids, and proteins. Within the TME of RCC, EVs may facilitate crosstalk among stromal, immune, and tumor cells, thereby potentially enhancing chemotherapy resistance, metastatic dissemination, and immune evasion [121]. Accumulating evidence indicates that RCC-derived EVs drive malignant progression through multiple distinct mechanisms: (1) Chemoresistance—EVs from drug-resistant RCC cells carry specific molecular cargo, such as the long non-coding RNA lncARSR, which enhances sunitinib resistance by functioning as a competing endogenous RNA in recipient cells [122]; (2) Angiogenesis Induction—RCC-secreted EVs deliver pro-angiogenic miRNAs, including miR-23a, to endothelial cells, where they stabilize HIF-1α by targeting Prolyl Hydroxylase Domain-containing protein 1, thereby stimulating tumor neovascularization [123]; (3) Immune Evasion—Tumor-derived exosomes express immunosuppressive molecules such as Programmed Cell Death-Ligand 1 (PD-L1) on their surface, which can bind to PD-1 on T cells, leading to inhibition of T cell activation and fostering an immunosuppressive microenvironment [124].

### 8.4 Potential Strategies for Regulating Mitochondrial Function in RCC Treatment

Given the pivotal role of mitochondrial function in RCC, targeting mitochondrial dysfunction holds significant clinical promise. A variety of agents modulating mitochondrial processes have been investigated, including herbal monomers, natural compounds, small-molecule inhibitors, and gene therapy approaches. Table 2 summarizes the primary therapeutic strategies currently under investigation, with detailed mechanisms and outcomes discussed in Section 9, Section 10, Section 11 and Section 12.

**Table 2:** Therapeutic strategies targeting mitochondrial dysfunction in RCC.

Therapeutic Strategy	Materials/Agents	Sources/Types	Research Type (Model Systems)	Key Targets & Effects (↑ Increase, ↓ Decrease)	Proposed Mechanisms	Main Outcomes	Tested Concentration/Dosage	References
Herbal Monomers	Ginsenoside Rg3	Panax ginseng	*In* *vitro* (786-O, ACHN cells)	↑ H3K9ac, ↑ H3K27ac, ↓ DNMT1, ↓ DNMT3a, ↓ DNMT3b, ↑ Caspase-3, ↑ Bax, ↓ Bcl-2	Promotes histone acetylation and DNA demethylation; Induces mitochondrial apoptosis	Inhibited RCC cell migration, invasion, colony formation, and tube formation; Enhanced apoptosis	*In* *vitro*: 0, 25, 50, 100 μM	[125]
	Cryptotanshinone	Salvia miltiorrhiza	*In* *vitro* (786-O, Caki-1 cells); *In vivo* (BALB/c nude mice)	↓ p-STAT3 (Tyr705), ↓ Cyclin D1, ↓ Bcl-2, ↓ Survivin, ↑ Cleaved Caspase-3, ↑ PARP cleavage	Suppresses STAT3 signaling pathway; Induces cell cycle arrest and mitochondrial apoptosis	Inhibited RCC cell proliferation and tumor growth; Induced G0/G1 phase cell cycle arrest and apoptosis	*In* *vitro*: 0, 2.5, 5, 10 μM; *In vivo*: 10 mg/kg	[126]
	Berberine	Coptis chinensis	*In* *vitro* (786-O, ACHN cells)	↑ ROS, ↑ γ-H2AX, ↓ Bcl-2, ↑ Bax, ↑ Cleaved Caspase-3, ↑ Cleaved PARP	Induces OS and DNA damage; Triggers mitochondrial apoptosis	Inhibited RCC cell proliferation, migration, and invasion; Induced G2/M phase cell cycle arrest and apoptosis	*In* *vitro*: 0, 25, 50, 100 μM	[127]
Natural Compounds	Quercetin	Widely in fruits & vegetables	*In* *vitro* (786-O, Caki-1 cells)	↑ TP53/p53, ↑ p21, ↓ Cyclin D1, ↓ CDK4, ↓ MMP-2, ↓ MMP-9	Regulates TP53 gene expression; Induces G1 phase cell cycle arrest; Inhibits MMP activity	Inhibited proliferation and migration of clear cell RCC cells	*In* *vitro*: 0, 25, 50, 100 μM	[128]
	Silibinin	Silybum marianum (Milk Thistle)	*In* *vitro* (Caki-1, ACHN, 786-O cells)	↑ Caspase-3/7 activity, ↓ Survivin, ↓ p-EGFR, ↓ p-ERK	Activates caspase cascade; Down-regulates anti-apoptotic protein; Blocks EGFR-ERK survival signaling	Inhibited RCC cell growth and induced apoptosis	*In* *vitro*: 50–200 μM	[129]
Small-Molecule Inhibitors	ABT-737 (in combination with Chloroquine)	Bcl-2 inhibitor (synthetic)	*In* *vitro* (786-O, ACHN cells)	↓ Bcl-2, ↓ Bcl-xL, ↑ Caspase-3 cleavage, ↑ PARP cleavage, ↑ LC3-II (inhibited by CQ)	Antagonizes Bcl-2/Bcl-xL to promote mitochondrial apoptosis; Chloroquine blocks compensatory autophagy	Synergistically induced apoptotic cell death in RCC cells	*In* *vitro:* ABT-737 (0.1–10 μM), Chloroquine (10 μM)	[130]
Gene Therapy	p53 Pathway Reactivation	Synthetic/Viral Vectors	Review Article (Preclinical models)	↑ p53, ↑ p21, ↑ Bax, ↑ PUMA, ↓ Bcl-2, ↓ MDM2/MDMX	Restores wild-type p53 tumor suppressor function; Induces cell cycle arrest and mitochondrial apoptosis; Overcomes resistance to conventional therapy	Suppresses tumor growth and progression; Enhances sensitivity to chemotherapy and targeted therapy in RCC	N/A (Therapeutic Strategy)	[131]

Abb: Bax, Bcl-2-associated X protein; Bcl-2, B-cell lymphoma/leukemia-2; Bcl-xL, B-cell lymphoma-extra large; CDK4, cyclin-dependent kinase 4; CQ, chloroquine; Cyclin D1, G1/S-specific cyclin D1; DNMT1, DNA methyltransferase 1; DNMT3a, DNA methyltransferase 3 alpha; DNMT3b, DNA methyltransferase 3 beta; EGFR, epidermal growth factor receptor; ERK, extracellular signal-regulated kinase; H3K9ac, histone H3 lysine 9 acetylation; H3K27ac, histone H3 lysine 27 acetylation; LC3-II, microtubule-associated protein 1 light chain 3-II; MDM2, murine double minute 2; MDMX, murine double minute X; MMP-2, matrix metalloproteinase 2; MMP-9, matrix metalloproteinase 9; N/A, not applicable; OS, oxidative stress; p21, cyclin-dependent kinase inhibitor 1A; PARP, poly(ADP-ribose) polymerase; p-STAT3 (Tyr705), phosphorylated signal transducer and activator of transcription 3 at tyrosine 705; PUMA, p53-upregulated modulator of apoptosis; RCC, renal cell carcinoma; ROS, reactive oxygen species; STAT3, signal transducer and activator of transcription 3; Survivin, baculoviral inhibitor of apoptosis repeat-containing 5; TP53/p53, tumor protein 53; γ-H2AX, phosphorylated histone H2AX.

## 9 Therapeutic Strategies Targeting Mitochondrial Dysfunction in RCC

Based on the five core mitochondrial processes discussed in Section 3, Section 4, Section 5, Section 6 and Section 7, therapeutic strategies targeting mitochondrial dysfunction in RCC can be categorized by their primary mechanisms of action. Below, we summarize representative agents according to the specific mitochondrial process they modulate.

### 9.1 Targeting Mitochondrial Energy Metabolism

Metabolic reprogramming in RCC involves a flexible interplay between glycolysis and OXPHOS, making both pathways potential therapeutic targets.

Ginsenoside Rg3, derived from Panax ginseng, promotes histone acetylation and DNA demethylation, leading to mitochondrial apoptosis and inhibition of RCC cell migration, invasion, colony formation, and tube formation (786-O, ACHN cells; 0–100 μM) [125].

Berberine, isolated from Coptis chinensis, triggers oxidative stress and DNA damage, activating mitochondrial apoptosis and suppressing RCC cell proliferation, migration, and invasion (786-O, ACHN cells; 0–100 μM) [127]. 

Quercetin, a flavonoid widely found in fruits and vegetables, regulates TP53 expression, induces G1 phase arrest, and inhibits MMP activity, suppressing proliferation and migration of clear cell RCC cells (786-O, Caki-1 cells; 25–100 μM) [132]. 

Silibinin, from milk thistle (Silybum marianum), activates caspase-3/7, downregulates Survivin, and blocks EGFR-ERK survival signaling, inhibiting RCC cell growth and inducing apoptosis (Caki-1, ACHN, 786-O cells; 50–200 μM) [129].

### 9.2 Targeting Mitochondrial Dynamics

Mitochondrial dynamics, particularly the balance between fusion and fission, influences tumor behavior and therapy response. Modulating this balance represents an emerging therapeutic avenue.

Cryptotanshinone, from Salvia miltiorrhiza, suppresses STAT3 signaling, inducing G0/G1 phase arrest and mitochondrial apoptosis, thereby inhibiting RCC proliferation *in vitro* (786-O, Caki-1 cells; 0–10 μM) and tumor growth *in vivo* (BALB/c nude mice; 10 mg/kg) [133].

### 9.3 Targeting Mitochondrial Apoptosis

Evasion of mitochondrial apoptosis is a hallmark of RCC. Restoring apoptotic pathways through Bcl-2 family inhibitors represents a direct therapeutic strategy.

The Bcl-2 inhibitor ABT-737, combined with chloroquine, antagonizes Bcl-2/Bcl-xL to promote mitochondrial apoptosis while chloroquine blocks compensatory autophagy, resulting in synergistic apoptotic cell death in RCC models (786-O, ACHN cells; ABT-737 0.1–10 μM, chloroquine 10 μM) [130].

p53 pathway reactivation using engineered viral or synthetic vectors restores wild-type p53 function, upregulating p21, Bax, and PUMA while downregulating Bcl-2 and MDM2/MDMX. This induces cell cycle arrest and mitochondrial apoptosis, overcoming conventional treatment resistance in preclinical RCC models [134]. 

## 10 Discussion

### 10.1 An Integrative Network of Mitochondrial Dysfunctions in RCC

A systematic examination of mitochondrial impairment mechanisms in RCC reveals an elaborate interactive network rather than isolated pathological events. These pathways engage in cross-talk, establishing a self-perpetuating cycle that promotes RCC advancement and treatment resistance.

As detailed in Section 3, Section 4, Section 5, Section 6 and Section 7, OXPHOS, mitochondrial dynamics, apoptosis, ROS, and mitophagy each exhibit context-dependent dual roles in RCC. This duality reflects the remarkable adaptive plasticity of mitochondrial function [107]. Both potentiation and suppression of mitochondrial activities can produce strikingly divergent, and sometimes opposing, biological outcomes, necessitating deeper exploration of the regulatory circuits that control mitochondrial behavior [37]. 

### 10.2 Driver versus Suppressor: Resolving the Central Question

Returning to the central question posed in the Introduction—whether mitochondrial dysfunction acts as a driver or a suppressor of RCC malignancy—the evidence reviewed in Section 3, Section 4, Section 5, Section 6 and Section 7 supports a context-dependent answer. Mitochondrial energy metabolism and dynamics predominantly function as drivers when reprogrammed to support proliferation, metastasis, and therapy resistance. Apoptosis evasion unequivocally acts as a driver by enabling uncontrolled cell survival. In contrast, ROS and mitophagy exhibit dual roles: low-to-moderate ROS or transient mitophagy promote adaptation and survival (driver), whereas excessive ROS or persistent mitophagy can trigger cell death (suppressor). Thus, the net effect depends on the specific pathway, signal intensity, and microenvironmental context, reframing the question as “under what conditions does each process drive or suppress malignancy?”

A critical analysis of this regulatory framework reveals three foundational concepts: (1) Mitochondrial bioenergetics is widely recognized as a fundamental driver of RCC pathogenesis [10]; (2) Mitochondrial apoptosis typically represents the convergence point of elaborate signaling networks that ultimately dictate cellular survival [135]; (3) Mitochondrial redox balance dynamically interfaces with various organellar functions, often instigating progressive decline associated with mitochondrial dysfunction [50]. These interconnected pathological mechanisms may collectively accelerate RCC progression.

### 10.3 Mitochondrial Fission: A Double-Edged Sword

The dual nature of mitochondrial fission—promoting cell death in treatment-sensitive contexts while reinforcing survival in resistant clones—warrants particular attention [29]. We hypothesize that nutrient availability in the TME (including glutamine scarcity) could dictate the functional consequences of fission by influencing Drp1 post-translational modifications. Future research should focus on delineating the complex interplay between mitophagy and mitochondrial bioenergetics, alongside clarifying the bidirectional regulation between mitophagy and apoptosis in RCC [29]. A systems-level understanding of these processes may provide a sophisticated conceptual paradigm for interpreting RCC pathobiology and formulating precision oncology strategies [136].

### 10.4 Upstream Drivers: Genetic Alterations, Regulatory Factors, and the TME

Emerging research has delineated three core mechanisms—genomic alterations, regulatory factors, and the TME—that underpin the aggressive phenotype of RCC and converge on mitochondrial activity [9,137]. Genomic instability (e.g., VHL, PBRM1 mutations), and epigenetic remodeling act as primary instigators of mitochondrial aberrations [79,93], fueling malignant traits [10,137]. A network of regulatory factors, including ncRNAs, p53, HSPs, sirtuins, and central metabolic regulators (PGC-1α, HIF-1α, PPAR-α, CPT1A), fine-tunes mitochondrial function and contributes to chemoresistance and metastasis [65,93,131,138,139,140]. The TME, through cellular components (TAMs, T lymphocytes, CAFs, adipocytes) and acellular factors (cytokines, EVs), imposes metabolic pressures that reshape mitochondrial function and promote tumor growth, immune evasion, and angiogenesis [141,142,143,144].

### 10.5 Clinical Translation: Therapeutic Opportunities and Challenges

In-depth exploration of mitochondria-related molecules holds clinical promise for RCC management, offering potential biomarkers for risk stratification and therapeutic targeting [9]. Recent years have witnessed a diversification of mitochondrial-targeting strategies for RCC treatment, encompassing natural compounds, synthetic small-molecule inhibitors, conventional chemotherapeutics, gene therapy, and nanotechnology applications [145,146]. Although these approaches show preclinical efficacy, their clinical translation faces key challenges: limited specificity, systemic toxicity, and resistance development often driven by adaptive metabolic shifts [146].

From a translational perspective, these strategies could be deployed at different disease stages [147]. Early-stage interventions might restore mitochondrial homeostasis; for advanced RCC, OXPHOS inducers or apoptosis inducers have shown promise [145,148]. Combining mitochondrial modulators with standard TKIs or immunotherapies may overcome adaptive resistance [146]. Future efforts should prioritize patient stratification using mitochondrial biomarkers to guide stage-specific and mechanism-based combination regimens [147,149].

Exemplifying these challenges, tthe Bcl-2 inhibitor ABT-737 causes dose-limiting thrombocytopenia, a toxicity that can be mitigated by nanocarrier-based delivery [150,151]. Sunitinib, while established in RCC treatment, can provoke severe adverse reactions [152]. Platinum-based therapies carry substantial toxicity and rapid resistance; natural compounds are being explored as adjuvants [153]. Gene therapy faces bottlenecks in safe and efficient delivery; nanoparticle systems offer promising solutions [154,155,156,157]. Natural compounds and traditional Chinese medicine monomers exhibit anti-RCC activity but are limited by poor bioavailability; advanced nanocarriers address instability and solubility issues [158,159]. Nanotechnology’s clinical implementation is hindered by the heterogeneous TME, multidrug resistance, and manufacturing complexity [160]. 

### 10.6 Future Perspectives: Emerging Technologies and Precision Oncology

While further clinical validation is needed, the evolving understanding of mitochondrial metabolism is fostering interdisciplinary precision therapeutics. High-throughput screening has identified novel inhibitors of carbonic anhydrase IX [161,162], and cryo-electron microscopy has resolved VHL-HIF architectures, enabling structure-based drug design [163]. Cutting-edge genomic and transcriptomic profiling (whole-genome sequencing, single-cell RNA-seq, spatial transcriptomics) is uncovering novel vulnerabilities. Functional precision medicine platforms (patient-derived organoids, ctDNA analysis) enable real-time assessment of drug sensitivity [161]. Integrating data from exceptional responder analyses and real-world evidence is accelerating biomarker discovery and clinical decision-making [164]. The synergistic implementation of these technologies is advancing personalized RCC management, with the potential to improve survival and patient-reported outcomes.

## 11 Critical Appraisal of the Context-Dependent Roles of Mitochondrial Processes in RCC

To move beyond descriptive duality toward mechanistic integration, it is essential to specify the precise conditions under which mitochondrial processes switch between pro-tumor and anti-tumor functions. Based on the evidence reviewed, three key determinants can be identified.

The first determinant is signal intensity or duration, which governs the outcomes of ROS and mitophagy. Available evidence suggests that ROS levels may follow a bell-shaped response: low-to-moderate ROS can activate adaptive survival pathways such as HIF-1α and nuclear factor kappa-B (NF-κB), promoting survival and proliferation. In contrast, excessive ROS appear to overwhelm cellular defenses, causing macromolecular damage and triggering apoptosis or ferroptosis [165,166]. Similarly, it is plausible that transient, low-level mitophagy serves a homeostatic function by removing damaged organelles, thereby supporting cell survival under mild stress [167]. Conversely, one might speculate that persistent or hyperactivated mitophagy could deplete the mitochondrial pool beyond a viable threshold, potentially leading to bioenergetic collapse and cell death.

The second determinant is the genetic and metabolic context, which dictates the role of OXPHOS. In VHL-deficient ccRCC, HIF-1α stabilization suppresses glucose oxidation, yet OXPHOS is sustained by alternative fuels such as glutamine or fatty acids [168]. Based on current experimental data, under nutrient-replete conditions, this may support proliferation (pro-tumor). However, it is conceivable that when glutamine is restricted or when complex I is pharmacologically inhibited, the same OXPHOS dependency could become a liability, potentially sensitizing cells to apoptosis (anti-tumor) [110]. This context-dependent vulnerability has been observed in ccRCC, where mitochondrial complex I activity is limiting for metastasis but not for primary tumor growth [110]. Thus, OXPHOS may act as a driver only when compensatory fuel sources are available.

The third determinant is the cellular spatial context, which determines the impact of mitochondrial fission. In therapy-sensitive cells, stress-induced Drp1 activation can lead to fragmentation and may facilitate apoptosis (anti-tumor). This interpretation is supported by observations linking this pro-apoptotic role of fission to ROS-dependent DRP1 activation, where excessive fission has been reported to trigger mitochondrial dysfunction and cell death [167]. In contrast, in resistant clones, emerging evidence suggests that the same fission machinery can be co-opted to generate metabolically fit fragments that localize to the leading edge of migrating cells, fueling invasion and metastasis (pro-tumor) [110,169]. The outcome might depend on the concomitant activation of survival pathways such as PI3K-AKT, which may override the pro-apoptotic consequences of fission [170].

In summary, the available evidence is consistent with the view that the switch between pro-tumor and anti-tumor functions could be governed by signal intensity (ROS/mitophagy), fuel availability (OXPHOS), and co-activated survival pathways (fission). If confirmed by future studies, recognizing these context-specific triggers moves the field from descriptive duality toward a predictive, mechanism-based framework.

## 12 Limitations

We acknowledge that the majority of the evidence discussed in this review is derived from preclinical studies, including *in vitro* cell line models and *in vivo* xenograft experiments. Furthermore, most studies have focused on ccRCC, leaving other RCC subtypes—such as pRCC and chRCC—underexplored [5,16]. Recent transcriptomic analyses have revealed that chRCC exhibits a distinct mitochondrial gene signature characterized by abundant abnormal mitochondria and downregulation of ETC components due to decreased mtDNA content, rather than complex I mutations [14,171]. Conversely, pRCC displays compromised oxidative phosphorylation and rewired glutathione metabolism [14]. These subtype-specific differences suggest that the generalizability of the discussed mitochondrial targeting strategies to non-ccRCC subtypes remains uncertain [62]. While the findings discussed herein provide important mechanistic insights into the role of mitochondrial dysfunction in RCC, their direct translation to clinical practice requires further validation in patient-derived samples, prospective clinical trials, and comparative studies across RCC subtypes. Therefore, the therapeutic implications discussed should be interpreted with caution.

## 13 Conclusion

In this review, we synthesize current knowledge on how mitochondrial dysfunction contributes to renal cell carcinoma (RCC), with a focus on its impact on tumor development, disease advancement, and treatment failure. Across five key areas—bioenergetics, organelle dynamics, programmed cell death, redox control, and selective mitophagy—the available evidence points to an interconnected regulatory network rather than a set of discrete events. Notably, mitochondrial activities often display opposing effects depending on the context: fission, ROS signaling, and mitophagy can either promote survival or facilitate elimination, influenced by signal strength, nutrient availability, and spatial cues. This contextual plasticity challenges rigid classifications and emphasizes the importance of mechanism-based stratification in both laboratory studies and clinical trial design.

The growing recognition of mitochondrial weaknesses has prompted the exploration of various interventions, including natural products, targeted inhibitors, gene-based approaches, and nanocarrier platforms. Nonetheless, major hurdles such as inadequate selectivity, adverse effects, and resistance arising from metabolic reprogramming continue to impede clinical translation. Mitochondrial impairment stands as a defining feature of RCC biology and a viable avenue for therapeutic innovation. Advancing the field will require a deeper understanding of mitochondrial–microenvironment crosstalk, subtype-specific metabolic traits, and the effective translation of fundamental discoveries into strategies that meaningfully enhance patient care and survival.

## Data Availability

No datasets were generated or analysed during the current study.

## Abbreviations

RCC	Renal Cell Carcinoma
ROS	Reactive oxygen species
ccRCC	Clear cell Renal Cell Carcinoma
ICIs	Immune checkpoint inhibitors
VHL	Von Hippel-Lindau
TME	The tumor microenvironment
mtDNA	Mitochondrial DNA
OXPHOS	Oxidative phosphorylation
ATP	Adenosine triphosphate
pRCC	Papillary RCC
chRCC	Chromophobe RCC
TCA	Tricarboxylic acid
ETC	Electron transport chain
OS	Oxidative stress
TKIs	Tyrosine Kinase Inhibitors
MDH2	Malate Dehydrogenase 2
IDH	Isocitrate Dehydrogenase
NADH	Nicotinamide Adenine Dinucleotide
SDH	Succinate dehydrogenase
SDHB	Succinate Dehydrogenase Subunit B
EMT	Epithelial-Mesenchymal Transition
OPA1	Optic Atrophy 1
OMA1	Overlapping Activity with m-AAA protease 1
STOML2/PHB2	Stomatin-like Protein 2/Prohibitin 2
Drp1	Dynamin-related protein 1
MFN2	Mitofusin 2
Bcl-2	B-cell lymphoma/leukemia-2
Bcl-xL	B-cell Lymphoma-extra Large
Mcl-1	Myeloid cell leukemia-1
HIFs	Hypoxia-inducible factors
GSH	Glutathione
GPX4	Glutathione Peroxidase 4
SOD2	Superoxide dismutase 2
MFF	Mitochondrial Fission Factor
HIF-1α	Hypoxia-Inducible Factor 1-alpha
BNIP3	Bcl-2/adenovirus E1B 19-kDa interacting protein 3
LC3-II	Microtubule-associated Protein 1 Light Chain 3-II
PGC-1α	Peroxisome Proliferator-activated Receptor Gamma Coactivator 1-alpha
CPT1A	Carnitine Palmitoyltransferase 1A
HSPs	Heat shock proteins
MMP	Mitochondrial membrane potential
TAMs	Tumor-associated macrophages
EVs	Extracellular vesicles
PD-L1	Programmed Cell Death-Ligand 1
